# Supplementary material for: Unexpected dynamics in femtomolar complexes of binding proteins with peptides
Source: Nat Commun. 2023 Nov 28;14:7823. doi: 10.1038/s41467-023-43596-2 (PMC10684580; doi:10.1038/s41467-023-43596-2)
Supplement: Supplementary file 1 — Supplementary Information [file 41467_2023_43596_MOESM1_ESM.pdf]

Supplementary information

Unexpected dynamics in femtomolar complexes of binding proteins with peptides

Stefano Cucuzza[1], Malgorzata Sitnik[1,4], Simon Jurt[1], Erich Michel[1,2], Wenzhao Dai[1], Thomas Müntener[3], Patrick Ernst[2], Daniel Häussinger[3], Andreas Plückthun[2]*, Oliver Zerbe[1]*

[1] Department of Chemistry, University of Zürich, Winterthurerstrasse 190, 8057 Zürich, Switzerland

[2] Department of Biochemistry, University of Zürich, Winterthurerstrasse 190, 8057 Zürich, Switzerland

[3] Department of Chemistry, University of Basel, St. Johanns-Ring 19, 4056 Basel, Switzerland

Supplementary Figures 1-15, Supplementary Table 1-2, Supplementary Dataset 1

Supplementary Figures:

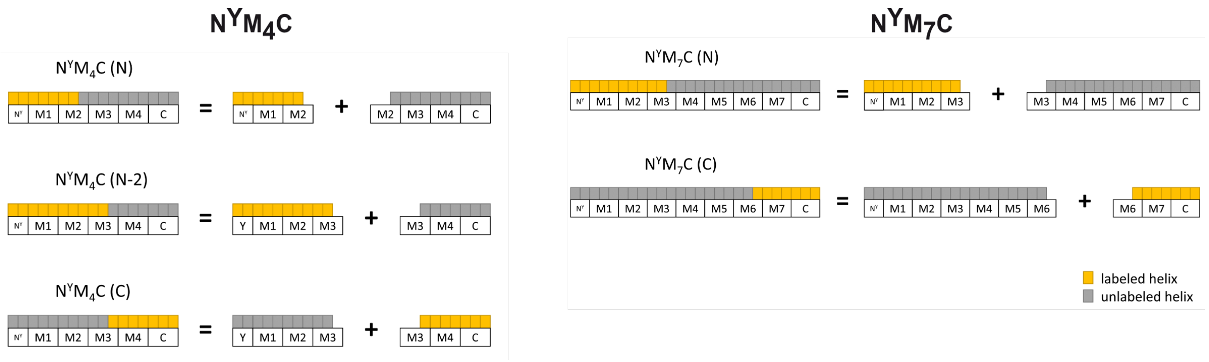

Supplementary Figure 1: Isotope labelling scheme used in this study. Each of the three helices is represented by a square. The N^Y and N^A cap contains only two helices. Yellow indicates ^{15}N and ^{13}C labelling, grey no labelling. Note that the central part of N^YM_7C (helix 3 of module 3, modules 4 and 5 and helices 1 and 2 of module 6) was never labelled, and thus it was impossible to assign residues from this region.

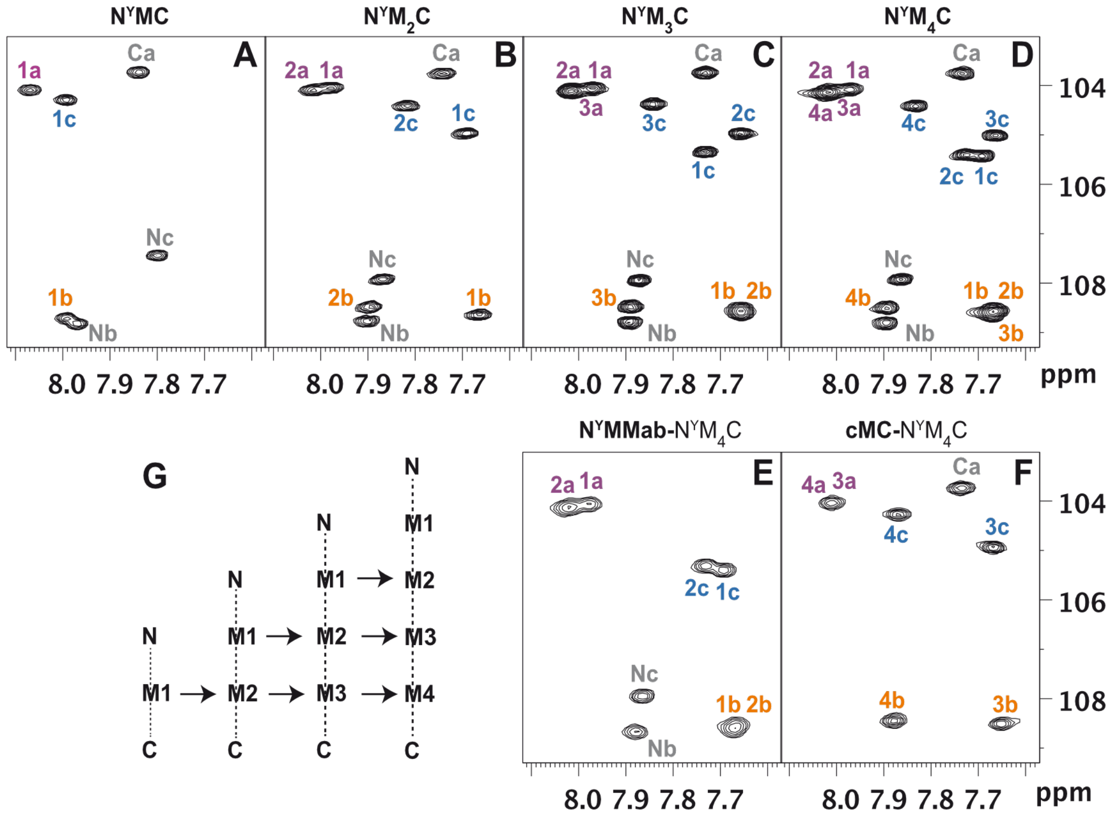

Supplementary Figure 2 Verification of assignment from N^{Y}M$_{n}$C proteins with a different number of internal modules. A-F Expansions of [^{15}N,^{1}H]-HSQC spectra show cross peaks from glycine residues. **G** Signals from modules close to the caps behave similarly and therefore can be traced as indicated in the scheme. E.g., signals from M1 in protein NMA are located at positions close to those of M4 in protein NM$_4$C.

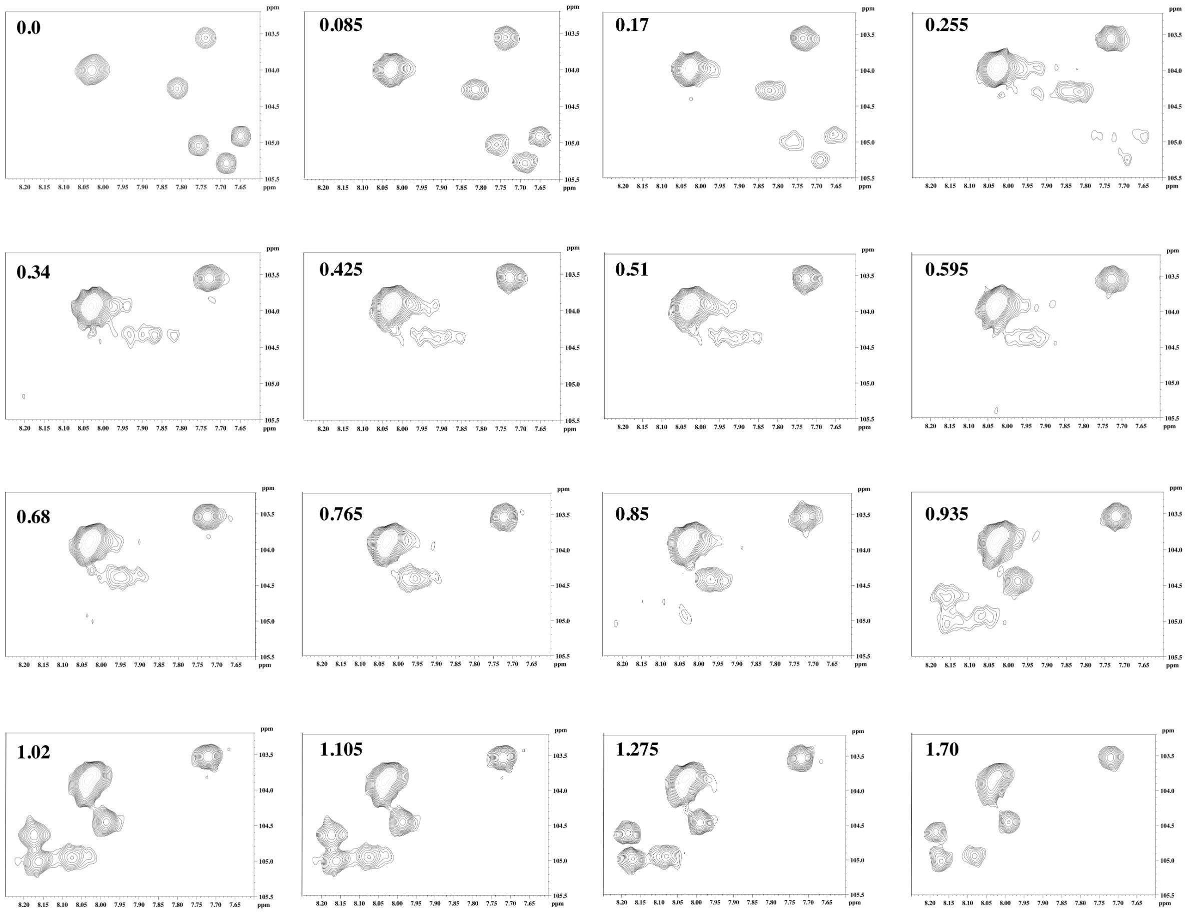

Supplementary Figure 3. Titrations of N^{A}M$_4$C. Expansions of the Gly c region of [^{15}N,^{1}H]-HSQC spectra of N^{A}M$_4$C with (KR)$_4$. The equiv. of added (KR)$_4$ is indicated in the top left of each spectrum.

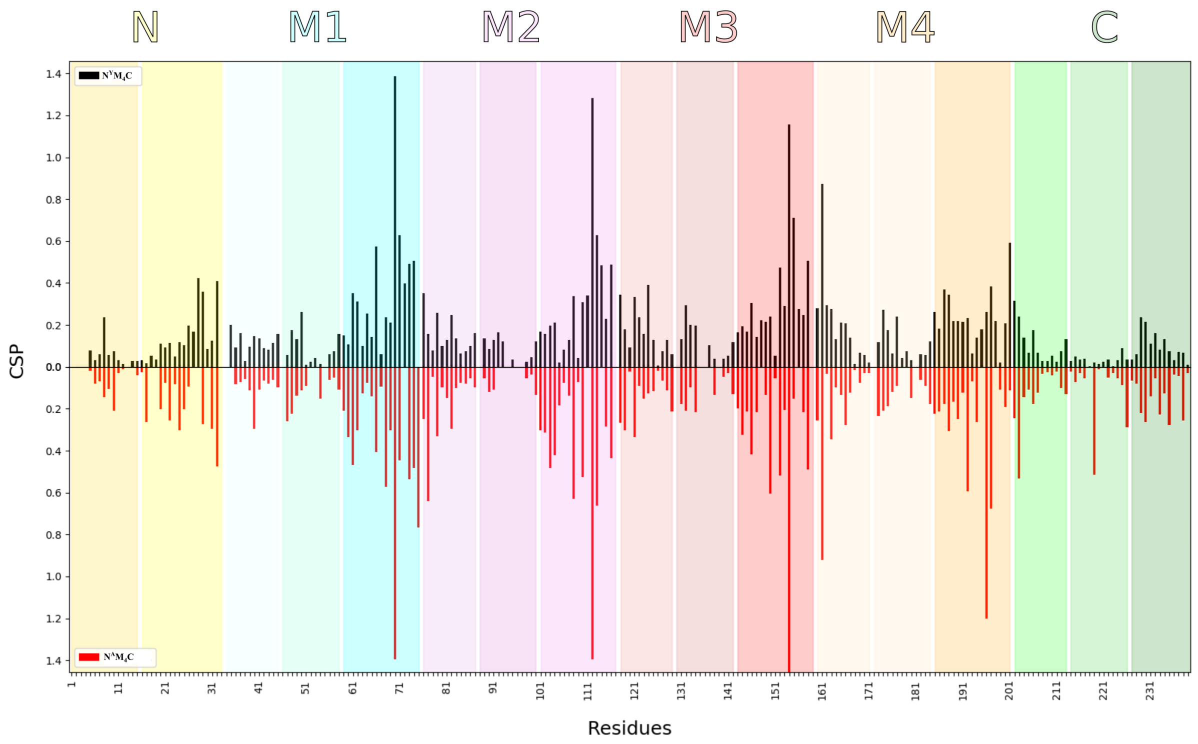

Supplementary Figure 4 Chemical shift perturbations (CSPs) of $N^{Y}M_4C$ (black) or $N^{A}M_4C$ (red) upon binding of $(KR)_4$. Undetermined CSPs, due to missing assignments, are set to a value of zero. A unique color is associated with each internal module or flanking terminal cap: N cap (yellow), M1 (cyan), M2 (purple), M3 (red), M4 (orange), C cap (green). Each of the three helices comprising an internal module or the C cap is indicated by a shade of the corresponding color. Note that the N^{Y} cap is composed by only two helices.

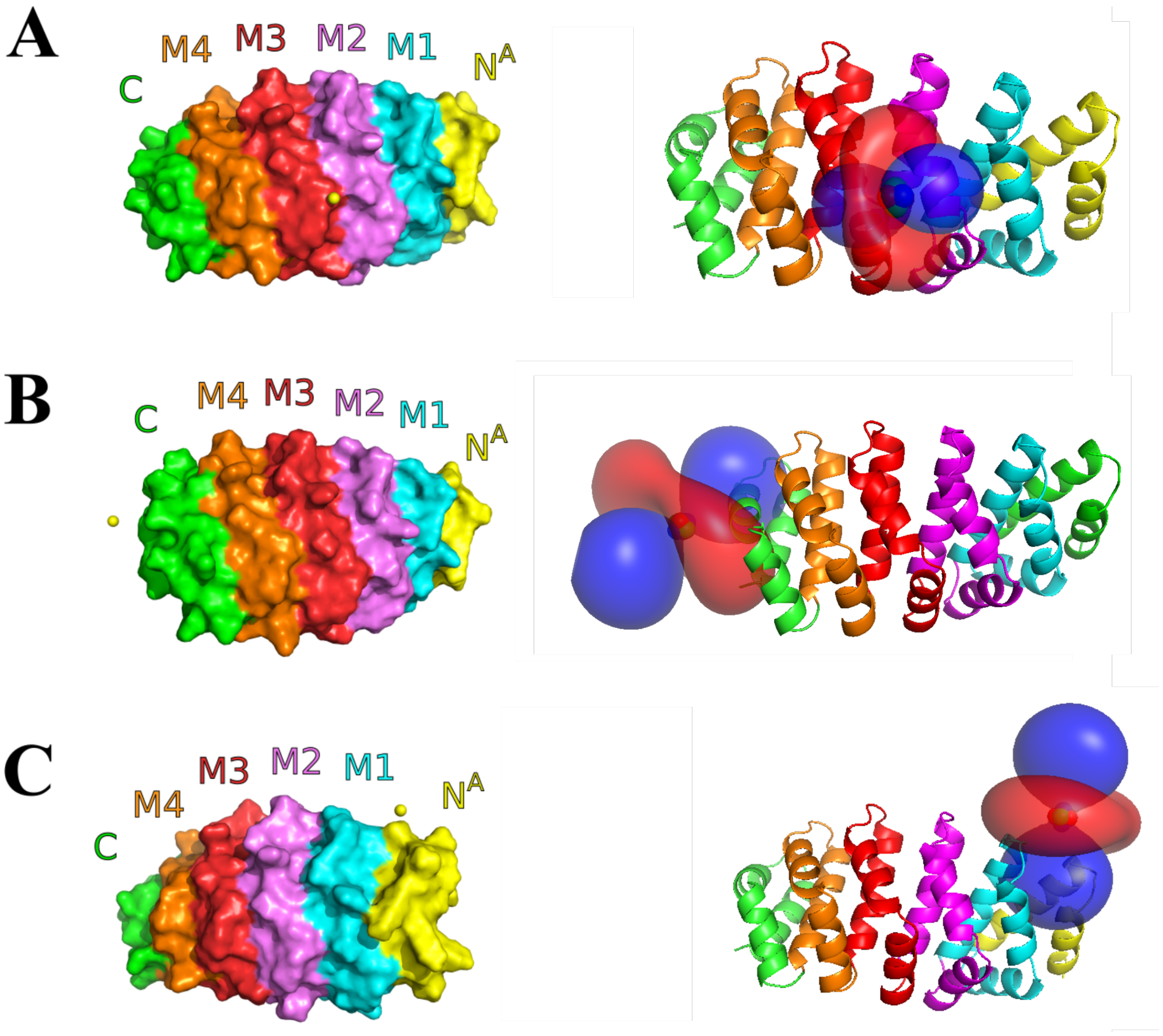

Supplementary Figure 5 N^{A}M$_4$C models coupled to the paramagnetic tag at different attachment sites. Model I (see Materials and Methods) was used as input structure to calculate the $\Delta\chi$-tensor components, using experimentally determined PCS values in the apo state. The thulium atom in the paramagnetic tag is represented as a yellow sphere while each N^{A}M$_4$C module and the caps are color-coded differently. On the left, the metal center position is depicted relative to the surface. On the right, the $\Delta\chi$-tensor is shown as an isosurface, with positive lobes depicted in blue and negative lobes depicted in red. **A** First attachment site, (M2)Q18C. **B** Second attachment site, (C)S21C. **C** Third attachment site, (N^{A})E15C. Isosurfaces are colored blue and red at 2.0 and -2.0 ppm (A), 2.0 and -2.0 ppm (B) and 3.0 and -3.0 ppm (C), respectively.

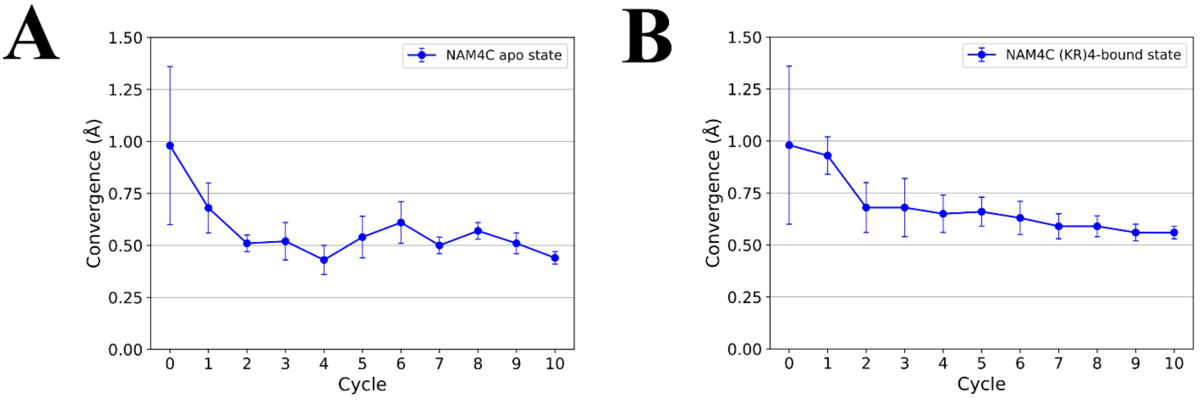

Supplementary Figure 6 Convergence of computed N^{A}M$_4$C structures as represented by the RMSD between structures calculated starting from the four models (see Materials and Methods). Error bars indicate the standard deviations (n = 4). **A** apo state. **B** (KR)$_4$-bound state.

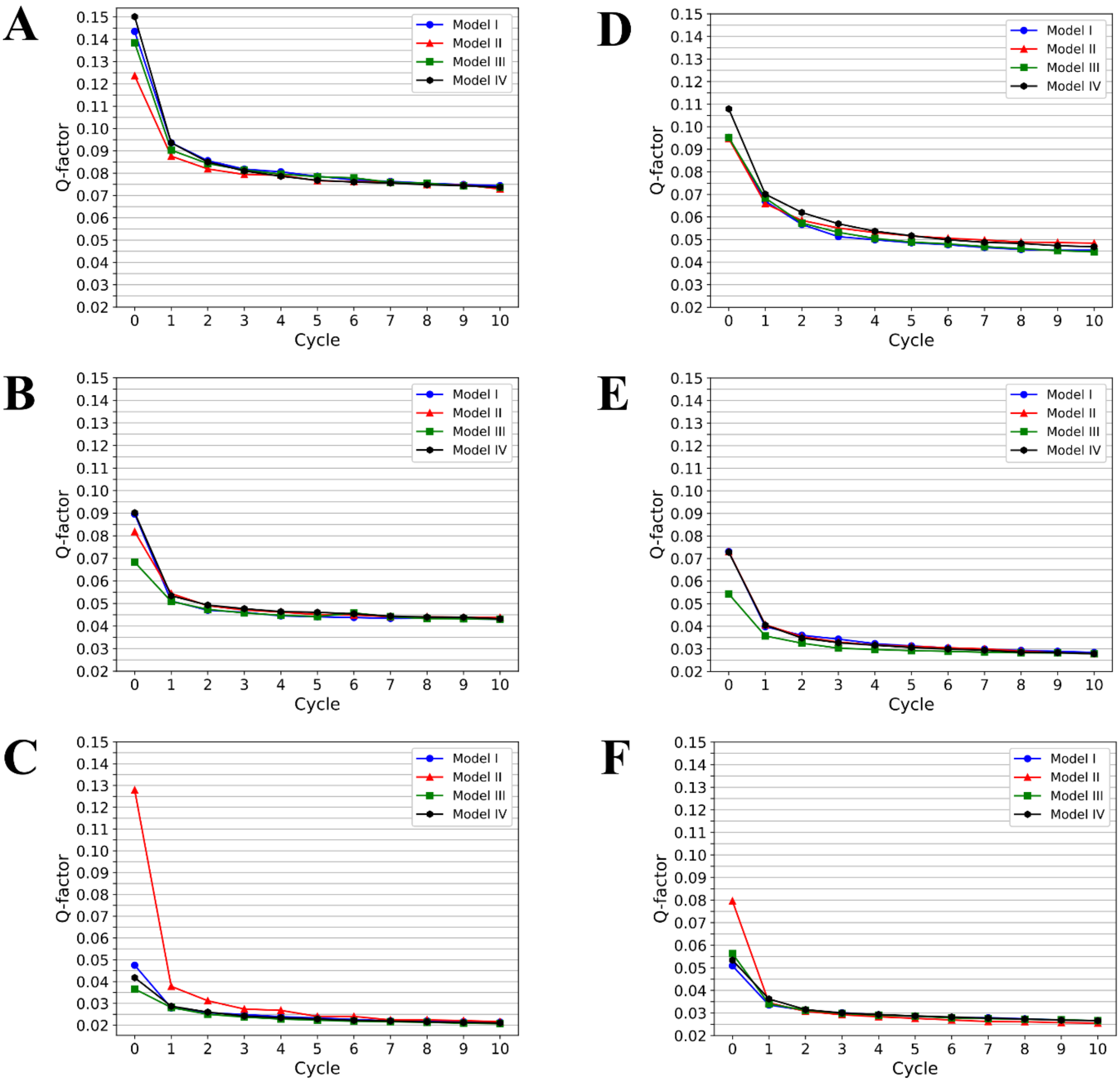

Supplementary Figure 7 Q-factors calculated with Paramagpy during the N^AM_4C refinement in the apo (panels **A**, **B** and **C**) or $(KR)_4$-bound states (panels **D**, **E** and **F**) for the three employed attachment sites. Each input model is depicted in a different series. The cysteine site is mentioned and the corresponding repeat in parentheses. **A** (M2)Q18C attachment site of the apo state. **B** (C)S21C attachment site of the apo state. **C** (N^A)E15C attachment site of the apo state. **D** (M2)Q18C attachment site of the $(KR)_4$-bound state. **E** (C)S21C attachment site of the $(KR)_4$-bound state. **F** (N^A)E15C attachment site of the $(KR)_4$-bound state.

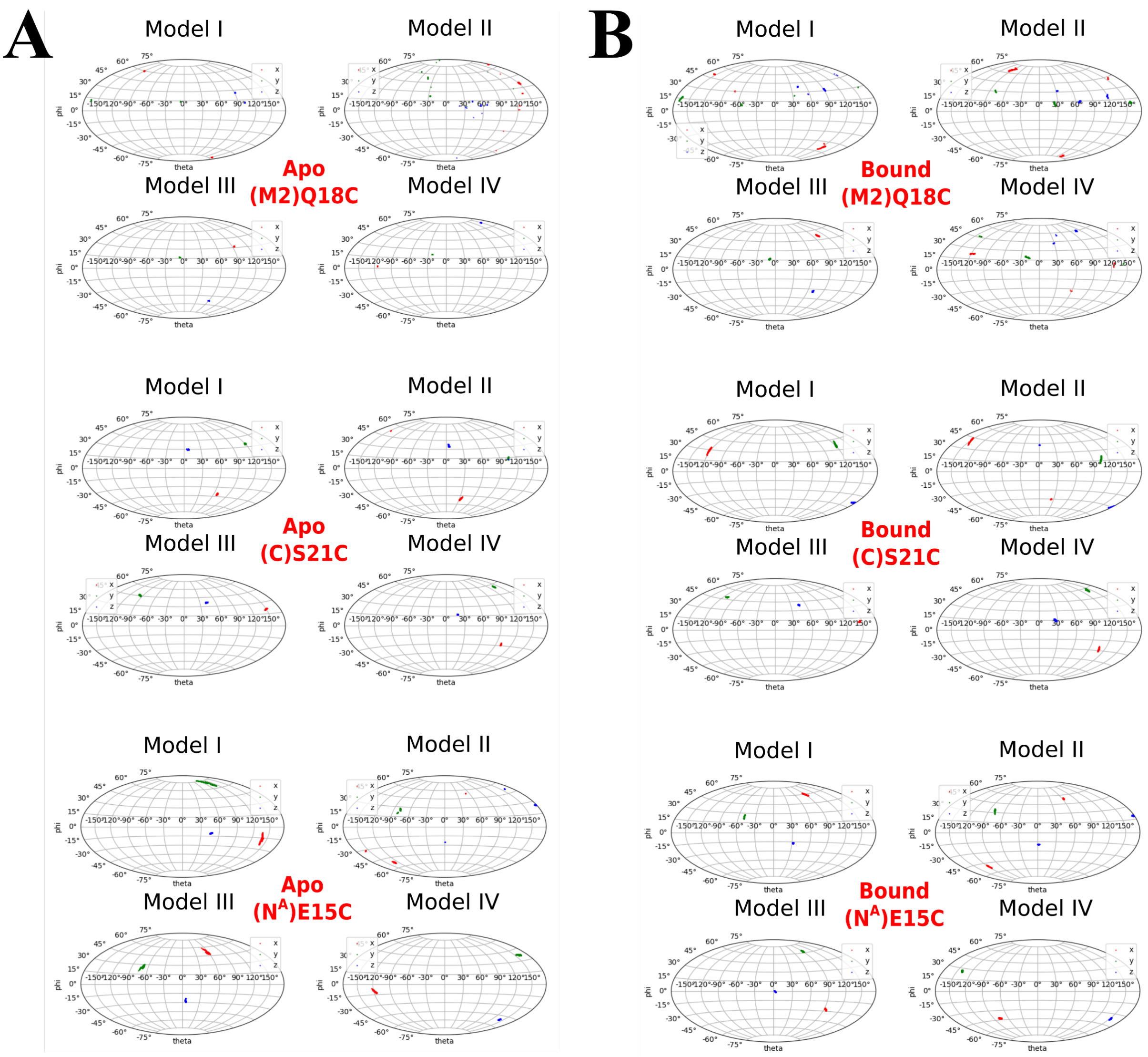

Supplementary Figure 8 Quality of N^{A}M$_4$C $\Delta\chi$-tensor fits estimated from a Monte-Carlo analysis performed with Paramagpy for the apo (left, **A**) and peptide-bound (right, **B**) states. The analysis was performed using single-fit mode through 200 iterations with an experimental uncertainty (σ) of 0.005 and 100% sample fraction, with the grid search centered on the x, y, z coordinates of the Cα atom of the attachment site. For each iteration, the resulting $\Delta\chi$-tensor's principal axis components are represented as a single dot in a Sanson-Flamsteed equivalent projection for the x- (red), y- (green) and z-axis (blue) components. For each attachment site, the fit was tested on the four available model structures I, II, III and IV (see Materials and Methods), at the very start of the iterative refinement (cycle 0). Shown are data for the first attachment site (M2)Q18C (top), second attachment site (C)S21C (center), and third attachment site (N^A)E15C (bottom).

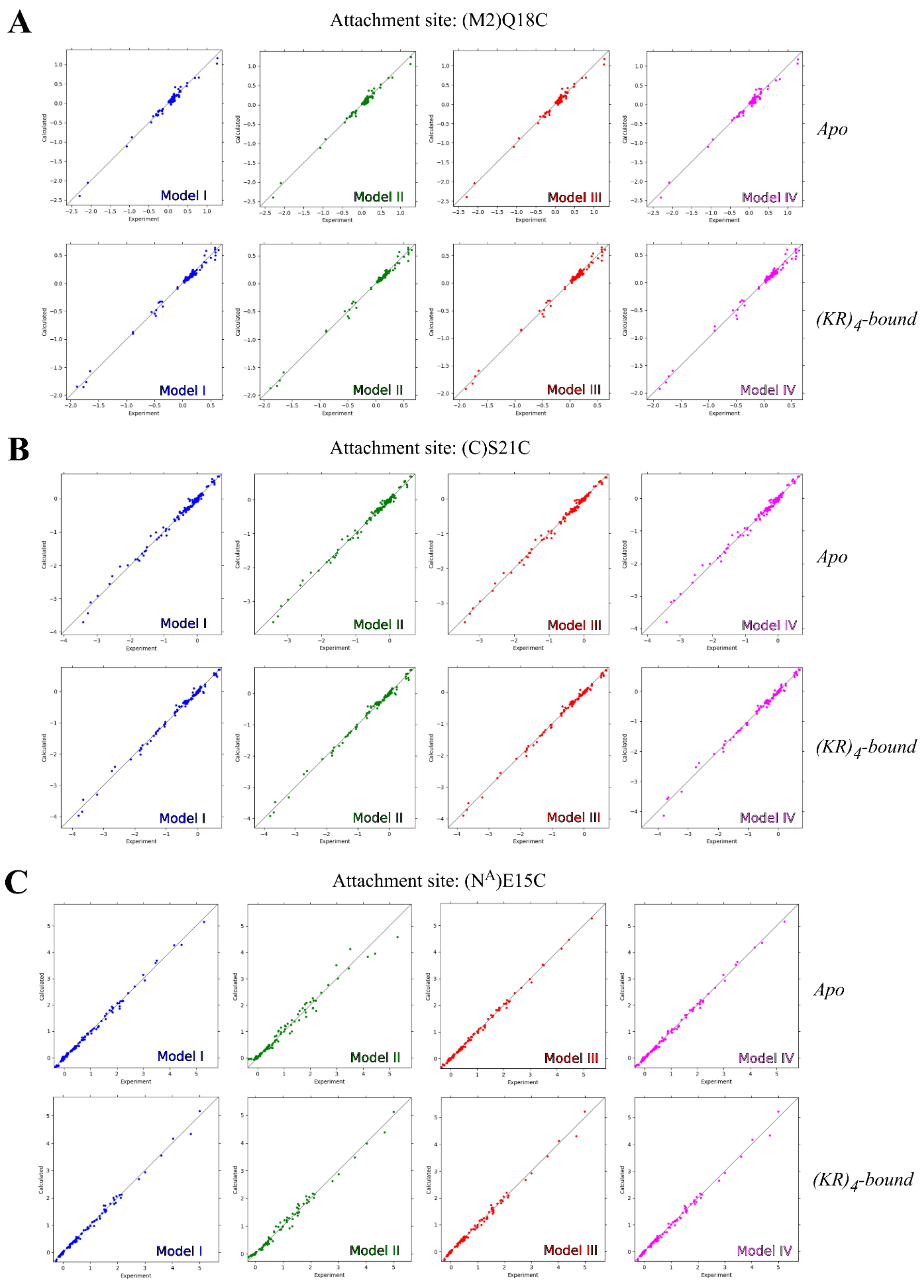

Supplementary Figure 9 Correlations between experimental and theoretical PCS back-calculated with Paramagpy from the initial four model structures (I-IV) used in the $N^{A}M_{4}C$ structure refinements for PCS data obtained from three differently tagged proteins in the apo (top) or $(KR)_4$-bound (bottom) states: **A** first attachment site, (M2)Q18C. **B** second attachment site, (C)S21C. **C** third attachment site, (N^{A})E15C.

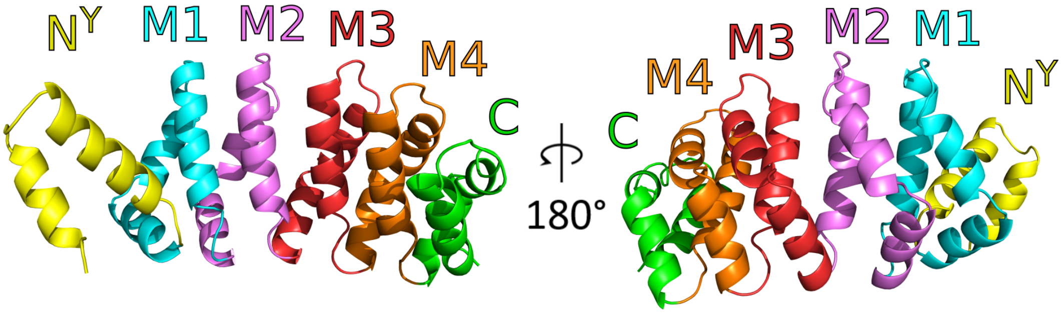

Supplementary Figure 10 Structure of the apo state of N^YM_4C, obtained by refinement through PCS (see Materials and Methods).

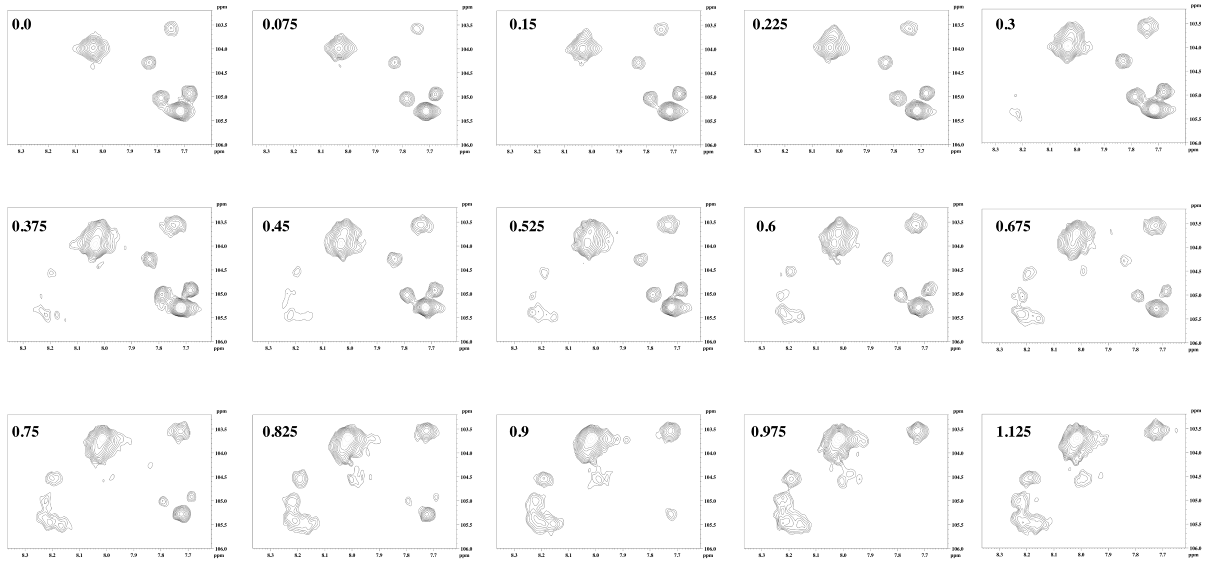

Supplementary Figure 11. Titrations of N^{A}M$_7$C. Expansions of the Gly region of [^{15}N,^{1}H]-HSQC spectra of N^{A}M$_7$C with (KR)$_7$. The molar equivalents of added (KR)$_7$ are indicated in the top left of each spectrum.

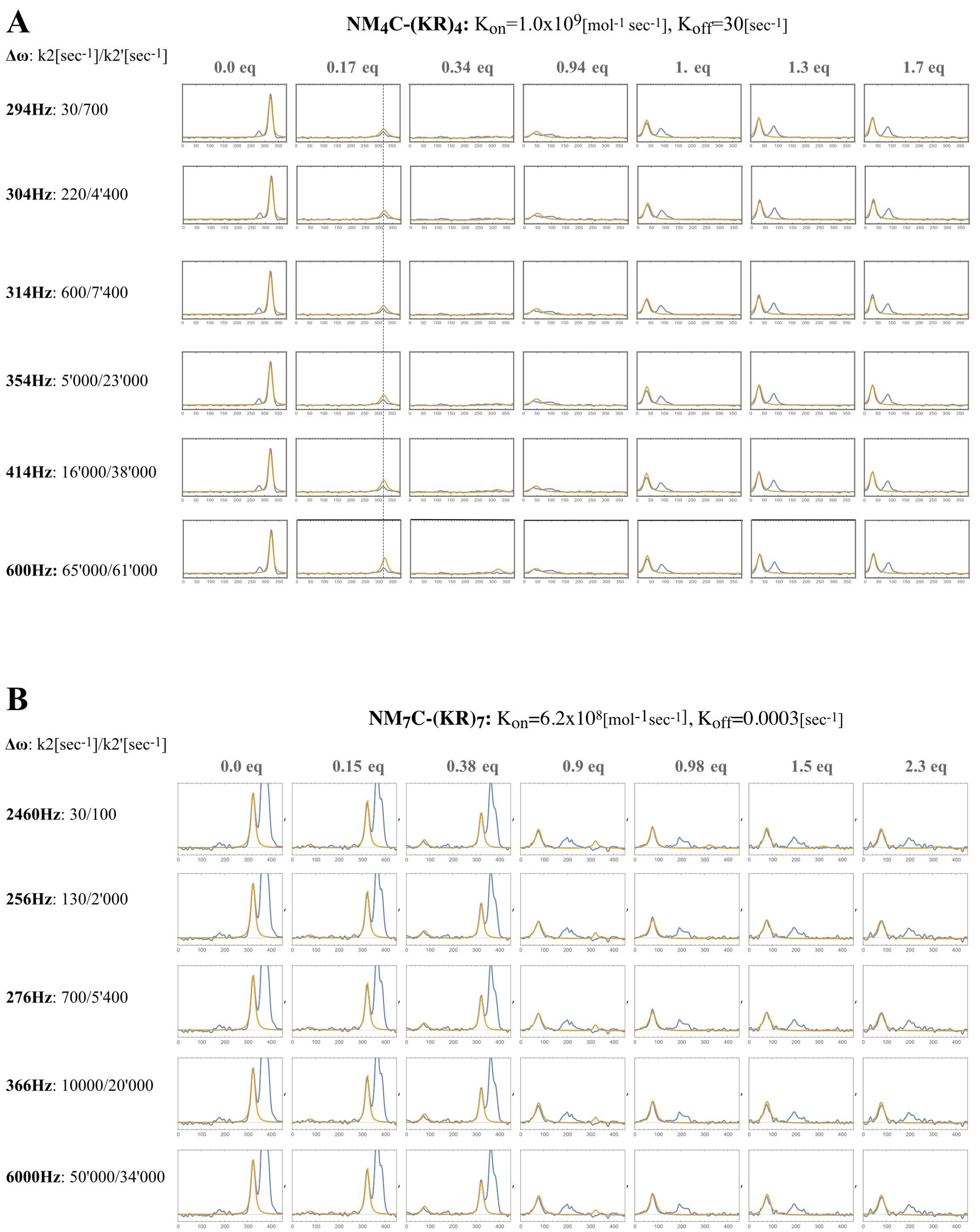

Supplementary Figure 12: Simulations of line-shapes and comparison with those obtained during the titration with peptides: (**A**) $N^{A}M_4C$-(KR)$_4$ or (**B**) $N^{A}M_7C$-(KR)$_7$. Simulations assumed a 2-step exchange process (see text) and varied the frequency separation between free and bound state. Values of k2 and k2' for best fits are indicated on the left. The top row corresponds to the frequency separation extracted from the spectra. In **A** peaks can be reasonably fit only when the true peak separation is close to the observed one, a particularly illustrative example is highlighted by the dashed line. In **B** exchange is too slow to see effects on free-state signals. The simulations demonstrate that the chemical shift of the bound state is close to the peak positions of the saturated state, at least for NM_4C-(KR)$_4$. Moreover, it seems reasonable that the chemical shifts of NM_7C-(KR)$_7$, that are very similar to those of NM_4C-(KR)$_4$, also represent the fully saturated state.

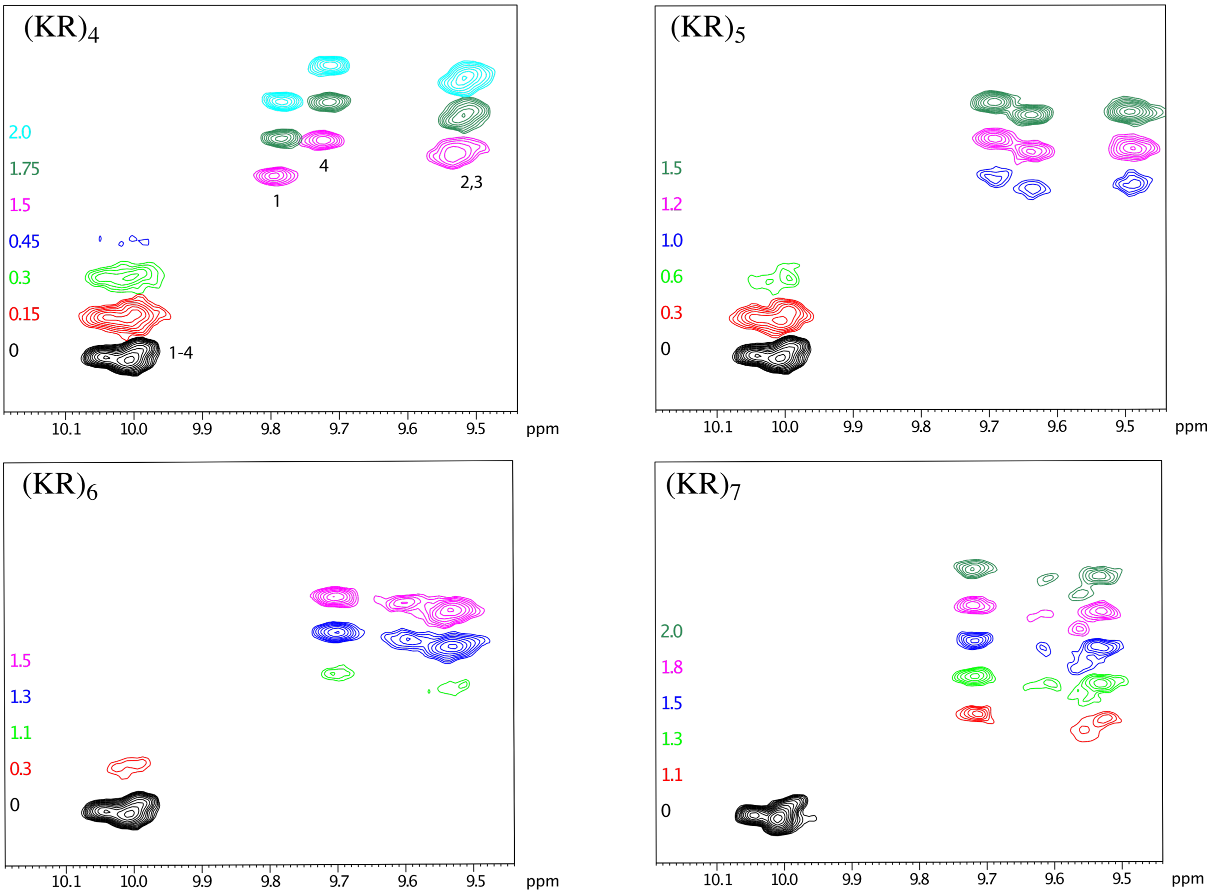

Supplementary Figure 13. Titrations of N^{A}M$_4$C. Expansions of the Trp indole region of [^{15}N,^{1}H]-HSQC spectra of N^{A}M$_4$C with (KR)$_4$ (top left), (KR)$_5$ (top right), (KR)$_6$ (bottom left) and (KR)$_7$ (bottom right). Spectra with different molar equivalents of peptides are color-coded and vertically shifted.

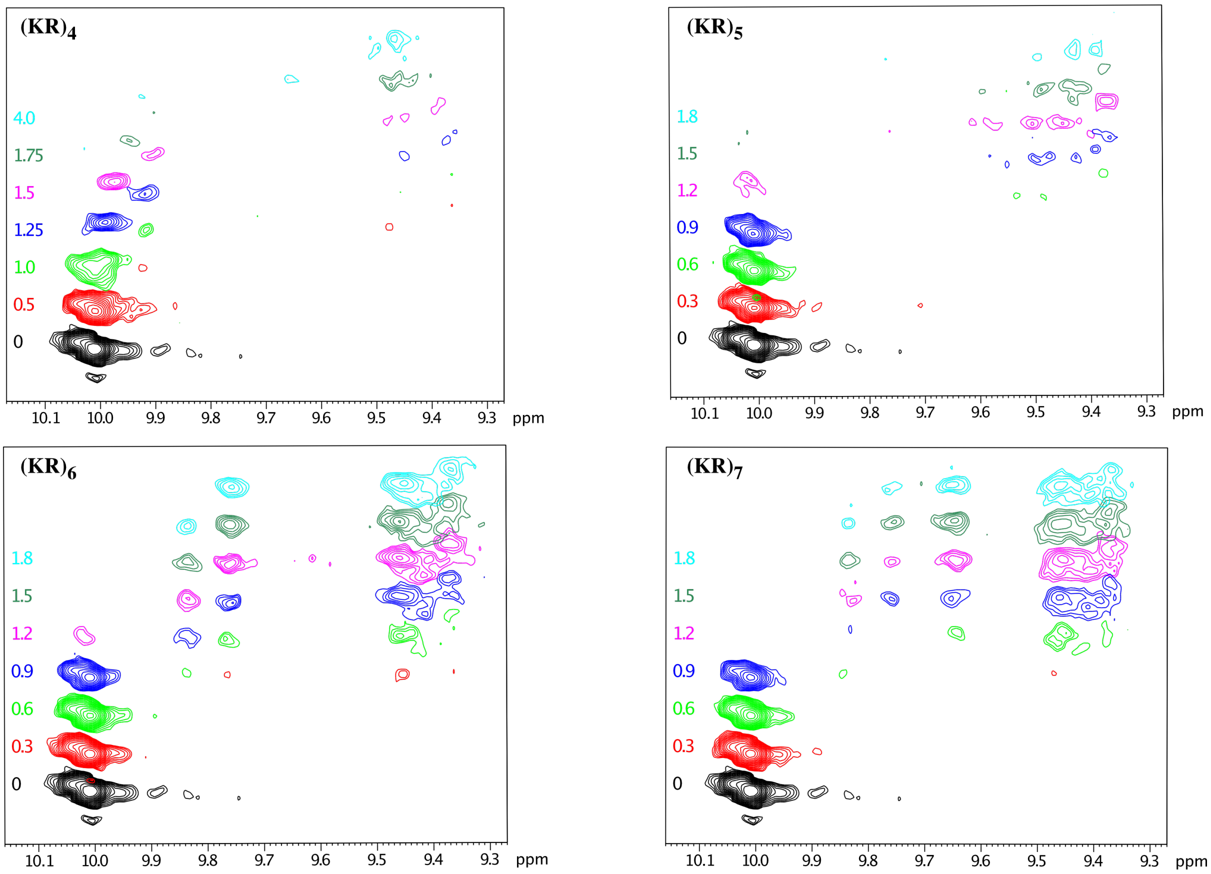

Supplementary Figure 14: Titration of N^{A}M$_7$C with (KR)$_n$-type peptides. Expansions of the Trp indole region of [^{15}N,^{1}H]-HSQC spectra of N^{A}M$_7$C with (KR)$_4$ (upper left), (KR)$_5$ (upper right), (KR)$_6$ (bottom left) and (KR)$_7$ (bottom right). Spectra with equiv. of peptides are color-coded and vertically shifted.

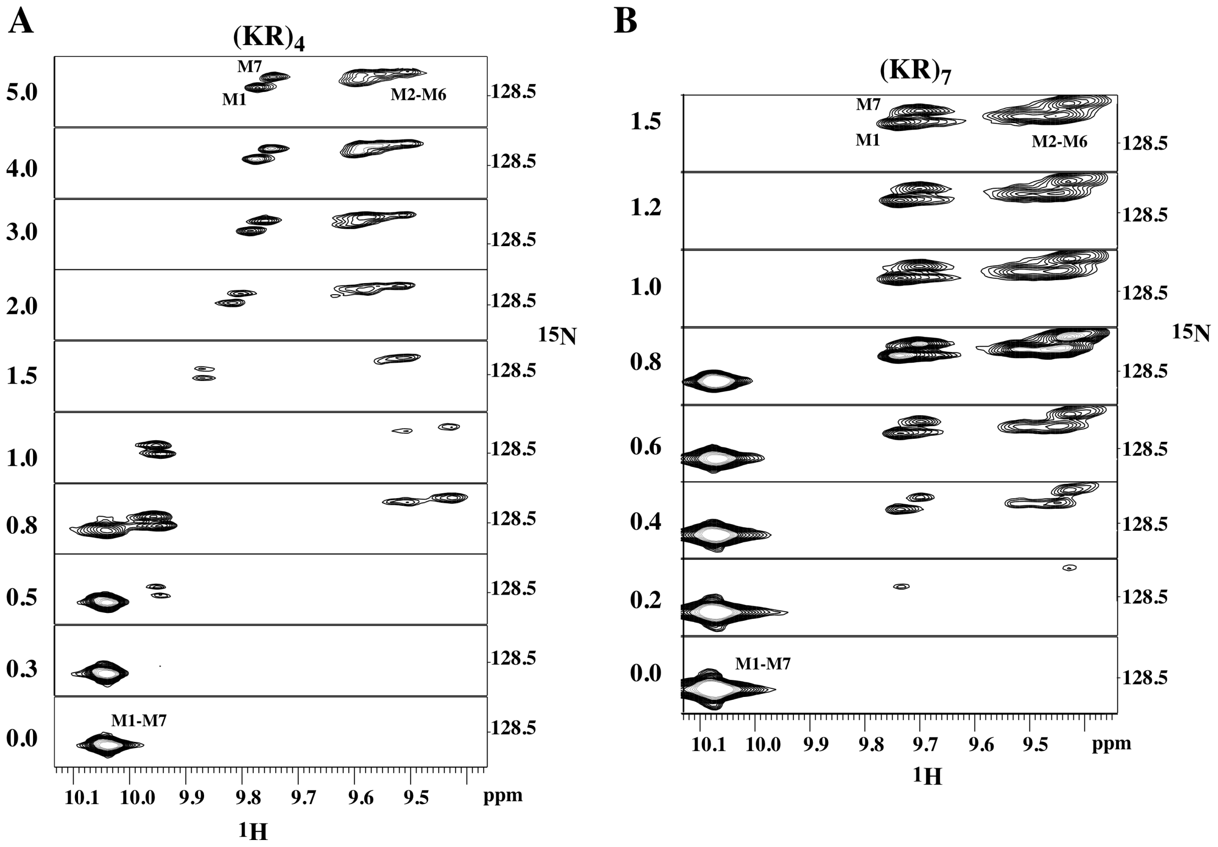

Supplementary Figure 15. Comparison of titrations of N^{Y}M$_7$C with (KR)$_4$ and (KR)$_7$. Expansions of the Trp indole region of [^{15}N,^{1}H]-HSQC spectra of N^{Y}M$_7$C with (KR)$_4$ (**A**) and (KR)$_7$ (**B**).

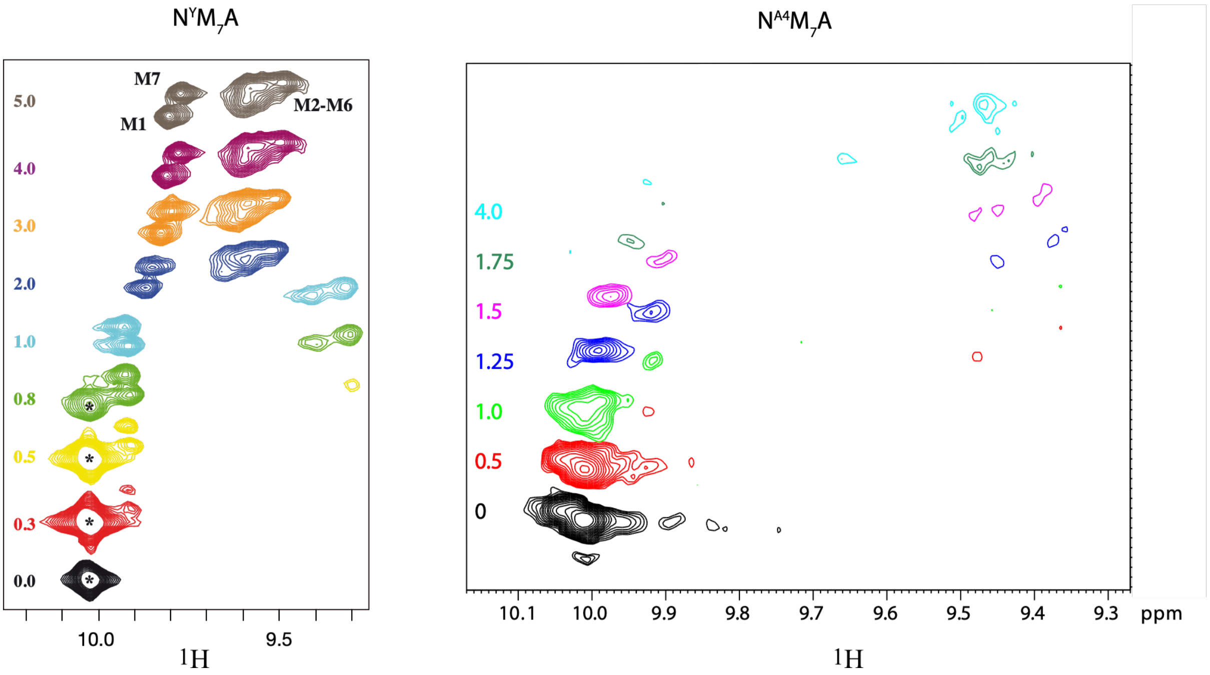

Supplementary Figure 16. Comparison of titrations of $N^{Y}M_7C$ (left) or $N^{A}M_7C$ (right) with $(KR)_4$. Expansions of the Trp indole region of $[^{15}N,^{1}H]$-HSQC spectra of NM_7C with $(KR)_4$. Spectra with equiv. of peptides are color-coded and vertically shifted.

Supplementary Tables

	RMSD (Å)	
	Apo	Bound
Model I	0.53	0.19
Model II	0.71	0.36
Model III	0.22	0.29
Model IV	0.10	0.22

Supplementary Table 1: Convergence with different seeds during the N^AM_4C refinement. RMSD is calculated between a structure after 10 cycles of refinement, starting from one of the four input models, and the structure undergoing the same procedure with a different seed value to validate the reproducibility of the process

Supplementary Table 2: NMR and refinement statistics for the structure refinement of N^AM_4C in the $(KR)_4$-bound state (note that *no NOEs were used*, see Methods section)

**NMR distance and dihedral constraints**	
Scaffold Distance constraints	1777
Total NOE	0
Intra-residue	
Inter-residue	
Sequential ($\|i-j\| = 1$)	
Medium-range ($\|i-j\| < 4$)	
Long-range ($\|i-j\| > 5$)	
Intermolecular	
Hydrogen bonds	
Total dihedral angle restraints	
ϕ	229
ψ	238
**Total PCS**	544
**Structure statistics**	
Violations (mean and s.d.)	
Distance constraints (Å)	0.06 ± 0.02
Dihedral angle constraints (º)	0.0 ± 0.0
Max. dihedral angle violation (º)	0.0 ± 0.0
Max. distance constraint violation (Å)	1.3 ± 0.7
Deviations from idealized geometry	
Bond lengths (Å)	0
Bond angles (º)	0
Impropers (º)	0
Average pairwise r.m.s. deviation** (Å)	
Backbone (res. 2-238)	0.51

** "Pairwise r.m.s. deviation was calculated among the 4 refined structures starting from 4 different model

All files used for calculating structures of free and$(KR)_4$-bound N^AM_4C have been deposited on Zonodo. This includes the starting models. Data can be accessed via the following link:

https://doi.org/10.5281/zenodo.8435468
