# Supplementary material for: Unexpected dynamics in femtomolar complexes of binding proteins with peptides
Source: Nat Commun. 2023 Nov 28;14:7823. doi: 10.1038/s41467-023-43596-2 (PMC10684580; doi:10.1038/s41467-023-43596-2)
Supplement: Supplementary file 3 — Description of Additional Supplementary Files [file 41467_2023_43596_MOESM3_ESM.pdf]

<u>Description of Additional Supplementary Files</u>

File Name: Supplementary Data 1

Description: Pseudocontact Shift Data

unliganded N^AM_4C

Sheet 2: Measured ^1H and ^{15}N PCS for attachment site Cys-18 (M2; residue 121)

Sheet 3: Measured ^1H and ^{15}N PCS for attachment site Cys-21 (C; residue 221)

Sheet 4: Measured ^1H and ^{15}N PCS for attachment site Cys-15 (N; residue 15)

Sheet 5: Attachment restraints for PCS tags (lower and upper limits from the corresponding Cα, from final cycle)

Sheet 6: Axial and rhombic components of PCS tensor for the three attachment sites in the 10 iterations . Axial and rhombic tensor components are given in 10^4*1 ppm * $Å^3 = 10^{-32}$ m^3.

(KR)₄-bound N^AM_4C

Sheet 7: Measured ^1H and ^{15}N PCS for attachment site Cys18 (M2; residue 121)

Sheet 8: Measured ^1H and ^{15}N PCS for attachment site Cys-21 (C; residue 221)

Sheet 9: Measured ^1H and ^{15}N PCS for attachment site Cys-15 (N; residue 15)

Sheet 10: Axial and rhombic components of PCS tensor for the three attachment sites in the 10 iterative cycles. Axial and rhombic tensor components are given in 10^4*1 ppm * $Å^3 = 10^{-32}$ m^3.

Sheet 11: Attachment restraints for PCS tags (lower and upper limits from the corresponding Cα)
